# Higher pre-treatment skin sympathetic nerve activity and elevated resting heart rate after chemoradiotherapy predict worse esophageal cancer outcomes

**DOI:** 10.1186/s12885-022-10180-8

**Published:** 2022-10-22

**Authors:** Chen-Ling Tang, Wei-Chung Tsai, Jui-Ying Lee, Yao-Kuang Wang, Yi-Hsun Chen, Yu-Wei Liu, Ming-Chieh Lin, Pen-Tzu Fang, Yu-Ling Huang, I-Chen Wu

**Affiliations:** 1 Division of Trauma and Acute Care Surgery, Department of Surgery, Kaohsiung Medical University Hospital, Kaohsiung Medical University, Kaohsiung, Taiwan; 2 Graduate Institute of Clinical Medicine, College of Medicine, Kaohsiung Medical University, Kaohsiung, Taiwan; 3 Department of Medicine, Faculty of Medicine, College of Medicine, Kaohsiung Medical University, Kaohsiung, Taiwan; 4 Division of Cardiology, Department of Internal Medicine, Kaohsiung Medical University Hospital, Kaohsiung Medical University, Kaohsiung, Taiwan; 5 Drug Development and Value Creation Research Center, Kaohsiung Medical University, Kaohsiung, Taiwan; 6 Division of Chest Surgery, Department of Surgery, Kaohsiung Medical University Hospital, Kaohsiung Medical University, Kaohsiung, Taiwan; 7 Division of Gastroenterology, Department of Internal Medicine, Sanmin Dist, Kaohsiung Medical University Hospital, Kaohsiung Medical University, No.100, Tzyou 1st Rd, Kaohsiung City, 80756 Taiwan; 8 Department of Surgery, Faculty of Medicine, College of Medicine, Kaohsiung Medical University, Kaohsiung, Taiwan; 9 Department of Internal Medicine, Kaohsiung Medical University Hospital, Kaohsiung, Taiwan; 10 Department of Radiation Oncology, Kaohsiung Medical University Hospital, Kaohsiung Medical University, Kaohsiung, Taiwan; 11 Department of Medical Imaging, Kaohsiung Medical University Hospital, Kaohsiung Medical University, Kaohsiung, Taiwan; 12 Center for Cancer Research, Kaohsiung Medical University, Kaohsiung, Taiwan

**Keywords:** Esophageal squamous cell carcinoma, Chemoradiotherapy, Resting heart rate, Skin sympathetic nerve activity, Autonomic nerve system, Survival

## Abstract

**Background:**

Chemoradiotherapy (CRT), which might affect the autonomic system, is the mainstay therapy for advanced esophageal squamous cell carcinoma (ESCC). Autonomic dysfunction has been found to possibly lead to cancer mortality in those with elevated resting heart rates (RHR). Skin sympathetic nerve activity (SKNA) is a new method of stimulating electrical signals in skin to evaluate autonomic function from sympathetic tone. In this study, we investigated the association between changes in RHR and autonomic function and ESCC mortality.

**Methods:**

Thirty-nine stage II-IV ESCC patients receiving CRT between March 2019 and November 2020 were prospectively enrolled and carefully selected, followed up and received the same meticulous supportive care. Serial RHR was recorded every two weeks from before CRT to eight weeks after CRT and average SKNA were recorded before and four weeks after CRT. All-cause mortality was defined as primary outcome.

**Results:**

We found the RHR of ESCC patients to be significantly elevated and peaking at four weeks after CRT (*p* < 0.001) and then to gradually decrease. Those with an elevated RHR above the cutoff level (18 beat-per-minute) at eight weeks after CRT had worse overall survival. In addition, those with higher baseline sympathetic tone (average SKNA ≥ 0.86 μV) also had poor outcome.

**Conclusions:**

Increased pre-treatment sympathetic tone and elevated RHR after CRT are alarm signs of poor ESCC outcome. Further exploration of the mechanisms underlying these associations could potentially lead to intervention strategies for reducing mortality.

**Trial registration:**

This study is registered with ClinicalTrials.gov, identifier: NCT03243448.

**Supplementary Information:**

The online version contains supplementary material available at 10.1186/s12885-022-10180-8.

## Introduction

In recent decades, elevated resting heart rate (RHR) has been identified as an important predictor for all-cause mortality, cardiovascular mortality, and cancer mortality [1–5]. RHR is also a predictor of death in some cancers such as gastrointestinal, colorectal, pancreatic, and non-small cell lung types [5, 6].

Although the relationship between RHR and cancer mortality is complex and not totally understood, some factors including autonomic imbalance, genetic factor, anticancer therapy, and physical activity are considered responsible for it [1, 2, 6]. Autonomic dysregulation is thought to be one of the most important factors. RHR reflects the balance between vagal and sympathetic systems [7], with elevated RHR indicating increased sympathetic activation and autonomic dysregulation [1, 6, 8]. Sympathetic nerve activation is linked not only to tumorigenesis, tumor proliferation and metastasis but also to arrhythmogenesis and cardiovascular events [1, 9, 10], hence, the sympathetic system might influence heart rate and tumor mortality simultaneously while on the other hand, the severity of tumors also reflects on sympathetic tone. Besides, Groarke et al. showed radiotherapy was also associated with elevated RHR and heart rate recovery and implied that was the result of radiotherapy-related autonomic dysfunction [11].

Skin sympathetic nerve activity (SKNA) analyzed by the neuECG recording is a novel method to harvest the electrical signal from the skin of subjects and can simultaneously record SKNA and electrocardiogram (ECG). neuECG is used as the sympathetic recording in several cardiac arrhythmic situations and might predict clinical outcome [12–14], so we intended to use SKNA as a novel biomarker to predict the outcome of our subject.

Esophageal cancer ranks as the sixth most common cancer worldwide and is the ninth leading cause of cancer mortality in Taiwan [15–17]. The main treatment of advanced esophageal cancer includes surgery, chemotherapy and chemoradiotherapy [17], with chemoradiotherapy (CRT) remaining the mainstay of treatment for advanced esophageal squamous cell carcinoma (ESCC). A previous study found Hodgkin’s type lymphoma patients who had received radiotherapy more than ten years previously had higher RHR than the general population [11]. In clinical practice, we also observed elevated heart rate and decreased blood pressure during CRT in our esophageal cancer patients.

However, to the best of our knowledge, no study has investigated the RHR and SKNA changes before and after CRT, nor their effects on oncology outcome. Hence, the present study aimed to access the heart rate and sympathetic tone changes before and after CRT for ESCC, determine the possible causes including autonomic system activity for RHR change, and investigate the interrelationship between RHR change and autonomic function and their impact on ESCC mortality.

## Material and methods

### Patient and study design

This study employs prospective data collection with retrospective analysis. Totally, 89 newly diagnosed, stage II-IV esophageal cancer patients were prospectively recruited between March 2019 and November 2020 at Kaohsiung Medical University Hospital. Participants were older than 20 years of age, had pathologically-diagnosed ESCC, stages II-IV disease by Eighth Edition AJCC of the performance status Eastern Cooperative Oncology Group (ECOG) 0–2, and had received CRT as the primary therapy. The exclusion criteria included those who had history of previous anti-cancer treatment or esophageal endoscopic submucosa dissection, with synchronous double primary cancer, cardiovascular disease, diabetes mellitus and those failing to complete CRT. Patients unwilling to participate in the neuECG study and those who lacked multiple SKNA data were excluded.

For planning of CRT protocol, patients were to receive radiotherapy 200 cGy/d for five days per week lasting for five weeks. The total dose of radiation was about 5000 cGy. Two cycles of chemotherapy with monthly cisplatin (75 mg/m^2^) on day 1 plus 5-fluorouracil (1000 mg/m^2^) day 1 to 4 were delivered every 28 days during radiotherapy. As for the role of CRT, the NCCN guideline suggests that radiation dose for esophageal cancer in preoperative and definite CRT to be 4140-5040 cGy and 5000-5040 cGy, respectively. In our hospital, a total dose of 5000 cGy is commonly used for both settings because some patients decline surgery after neoadjuvant CRT, and arranging add-on radiation can also be difficult. Therefore, our protocol is almost identical for neoadjuvant and definite CRT. This study is registered with ClinicalTrials.gov, identifier: NCT03243448. This study was approved by the Institutional Review Board (IRB) of Kaohsiung Medical University Hospital (KMUHIRB-E(𝐈)-20,190,051). All patients signed informed consents and were followed up until death or the end of this study in September 2021.

### Collection of clinical information and vital signs

The clinical stage of each patient was reviewed by our multidisciplinary esophageal cancer team and recorded to analysis. The primary tumor location and maximum tumor length by EGD or chest CT were also recorded. A serial of blood exams, treatment response, recurrence and survival information were obtained during patient follow-up.

RHR (bpm) and blood pressure (mmHg) were assessed by automatic blood pressure monitor before CRT and at every outpatient visit till two months after completion of CRT (Supplementary Fig. 1). In total, each participant had six measurements in a sitting position after resting for five minutes or more during a 16-week period. Autonomic system activity was accessed by SKNA and heart rate variability (HRV) in the last visit before CRT and four weeks after CRT (12th week).

### neuECG, SKNA and HRV

The detailed method of neuECG recording was modified from our previous study [14]. In brief, neuECG used conventional ECG electrodes in lead I configuration and equipment with a very high sampling rate (10,000 Hz) and wide sampling bandwidth (1–2000 Hz) version of MEGA ME6000 Biomonitor System (Mega Electronics Ltd, Finland) to harvest the electrical signal from the skin of subjects. The signals were then bandpass-filtered between 500 to 1000 Hz to display SKNA and between 1 to 150 Hz to display ECG. The neuECG recording was made during baseline, stress [18] and recovery phases (five minutes for each phase). The stress phase consisted of mental arithmetic stress induced by arithmetic problem-solving involving subtraction of serial 13’s from 1000 for five minutes. Data were analyzed to determine the average SKNA (aSKNA, µV) per digitized sample by a customized software. The study participants rested in the supine position for at least ten minutes before SKNA measuring in the ECG recording room and were asked to be still during SKNA measurement to avoid motion artifact. Average temperature and moisture were measured as around 23 ± 2 °C and 50 ± 5% in the recording areas. A research assistant blinded to the patients’ clinical status analyzed the SKNA pattern using customized software.

We used MATLAB (Mathworks, Inc., USA) based software heart rate variability analysis software (HRVAS) (https://github.com/jramshur/HRVAS) to analyze the HRV [19]. In brief, the R peak of QRS complex in ECG signal obtained by the neuECG was automatically detected by the modified Pan Tompkins algorithm, and the R-R interval was obtained beat-by-beat [20]. Time-domains of HRV were then calculated by Matlab and HRVAS. The standard deviation of normal-to-normal beat intervals (SDNN) was used to represent the five-minute time-domain HRV.

### Questionnaire

Our experienced research nurse interviewed the participants using a standard questionnaire to collect information on demographic and lifestyle factors [21]. We used the European Organization for Research and Treatment for Cancer (EORTC) QLQ-C30 questionnaire to access patients’ functional domains, symptoms and financial difficulty [22]. A disease-specific questionnaire for esophageal cancer (QLQ-OES18) was also used, with items in the questionnaire transformed to score 0 to 100, where higher scores equated to worse symptoms and better scores to functional scales.

### Statistical analysis

The distributions of demographic characteristics and clinical characteristics were presented as the means ± standard deviation (SD) or number (%). The differences of RHR, blood pressure and autonomic function during CRT were analyzed by the Student *t* test for continuous variables. Chi-square test was used to compare the changes in quality of life (QoL) score and CRT response between the two groups by heart rate change. Receiver operating characteristics curve was applied to calculate the cut-off value of average skin sympathetic nerve activity (aSKNA) and heart rate change. The cut-off values of aSKNA and heart rate change were the point on their ROC curve with minimum distance from the left-upper corner of the unit square-the point where the Youden’s index was maximum [23]. Overall survival analysis in the different groups used cox regression model to calculate hazard ratio and 95% confidence interval (CI) while log rank test was used to compare probability of survival in different groups. When using neuECG to obtain HRV results, there were some SDNN data missing during transformation. No matter the missing occurred in the phase before or after CRT, the patient would be excluded from the corresponding phase while comparing SDNN before and after CRT.

Given the structure of repeated measure data for serial RHR and blood pressure, we used generalized estimating equation (GEE) models to analyze the trends of changes in the two parameters among different groups. Data were analyzed using the Stata SE 15.0 (StataCorp, College Station, Tex), and *p* < 0.05 was considered significant. 

## Results

**Fig. 1:**
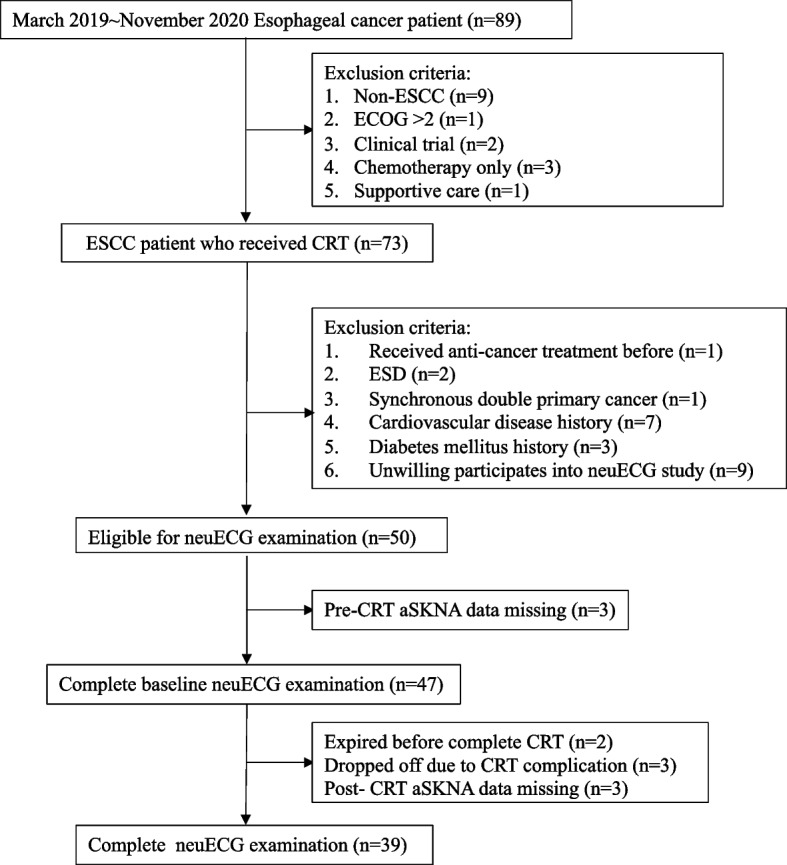
Selection process of study population Abbreviations ESCC: Esophageal squamous cell carcinoma, CRT: chemoradiotherapy, ESD: endoscopic submucosa dissection, SKNA: skin sympathetic nerve activity, HRV: heart rate variability^*^

Among the 89 eligible esophageal cancer patients, fifty patients had participated in the neuECG study for autonomic imbalance evaluation. After excluding those with multiple missing SKNA data, a total of 39 patients were analyzed in this study (Fig. 1).

### Baseline characteristics

The mean follow-up time of the 39 patients analyzed in this study was 16.10 ± 6.52 months, with the mean age being 59.15 ± 6.56 years while 18 (46.15%) were younger than 59 years (Table 1). Most of our patients had stage II-III diseases (61.54%).Five patients (12.82%) with small but suspicious lung nodules were diagnosed as metastatic disease. However, they were in good condition and had bulky primary tumors or regional lymph nodes. Radiation was therefore added to chemotherapy for better local control and to palliate dysphagia. Twenty-five (64.10%) patients with upper or mid-third esophageal cancer had a radiation field covering the stellate ganglion, while seventeen (43.59%) patients had a primary tumor longer than the median level (7 cm). The mean baseline BMI (body mass index) was 23.28 ± 3.28 kg/m^2^, mean baseline albumin level was 4.29 ± 0.38 g/dL, and neutrophil-to-lymphocyte ratio (NLR) was 3.72 ± 1.63. Most patients (84.6%) had received a total radiation dose of 5000-5040 cGy and 7–10% had a dose reduction in chemotherapy due to side effects (Table 1).

**Table 1:** Demographic and clinical information of the 39 esophageal squamous cell carcinoma patients who underwent HRV before and after CRT

Variable	N (%)
Age, years (mean ± SD)	59·15 ± 6·56
Sex, Male	36 (92·31%)
T stage	
2	9 (23·08%)
3	22 (56·41%)
4A	8 (20·51%)
N stage	
0	4 (10·26%)
1	35 (89·74%)
Overall stage	
2 & 3	24 (61·54%)
4	15 (38·46%)
Tumor location	
Upper & Middle	25 (64·10%)
Lower	14 (35·90%)
Tumor length^1^	
≤ 7 cm	21 (53·85%)
> 7 cm	17 (43·59%)
Missing	1 (2·56%)
Total cisplatin dose	
< 120 mg/m^2^	3 (7·69%)
≧120 mg/m^2^	36 (92·31%)
Total 5-FU dose	
< 6000 mg/m^2^	4 (10·26%)
≧6000 mg/m^2^	35 (89·74%)
Total radiation dose	
5000–5040 cGy	33 (84·62%)
> 5040 cGy	6 (15·38%)
Baseline BMI, kg/m^2^ (mean ± SD)	23·28 ± 3·28
Baseline albumin, g/dL (mean ± SD)	4·29 ± 0·38
Baseline NLR	3·72 ± 1·63
Alcohol, yes (%)	37 (94·87%)
Betel nut, yes (%)	26 (66·67%)
Smoking, yes (%)	35 (89·74%)
EORTC QLQ-C30 score (mean ± SD)	80·20 ± 10·97
EORTC QLQ-OES18 score (mean ± SD)	86·83 ± 5·15
CRT response	
Partial response	31
Stable and progressive disease	8
Operable after CRT, yes (%)	20 (51.28%)
Underwent operation after CRT, yes (%)	17 (43.59%)

*Abbreviations: HRV *Heart rate variability, *CRT *chemoradiation therapy, *SD *standard deviation, *cGy *centi-gray, *BMI *body mass index, *NLR *neutrophil-to-lymphocyte ratio, *EORTC QLQ C-30 *European Organization for Research and Treatment of Cancer quality of life questionnaire, *EORTC QLQ-OES18 *European Organization for Research and Treatment of Cancer quality of life questionnaire for oesophageal cancer

### RHR, SKNA, SDNN before and after CRT

Comparing the short-term change in RHR, blood pressure and aSKNA with the baseline data, only RHR was significantly elevated at four weeks after CRT (Table 2). The elevating trend of RHR after CRT remained significant in subgroup analysis by tumor stage, tumor site, tumor length (> 7 cm and $\le$ 7 cm) and BMI (< 24 and 24 ≤ BMI < 27). The radiation exposure of left stellate ganglion, right stellate ganglion, or superior vena cava and right atrial did not make significant difference in aSKNA and SDNN before and after CRT.

**Table 2:** Comparison of change in RHR, blood pressure and autonomic function before and after CRT

		Before CRT	4 weeks after CRT	
	n	Mean ± SE	Mean ± SE	***p* value**
RHR (bpm)				
	39	76·36 ± 1·81	92·52 ± 2·41	< 0·001
SBP (mmHg)				
	39	123·33 ± 2·58	123·21 ± 2·32	0·97
DBP (mmHg)				
	39	76·82 ± 1·52	74·10 ± 1·73	0·17
MAP (mmHg)				
	39	92·32 ± 1·69	90·47 ± 1·73	0·38
aSKNA				
baseline (μV)	39	0·77 ± 0·03	0·79 ± 0·03	0·59
stress (μV)	39	1·39 ± 0·08	1·32 ± 0·07	0·34
recover (μV)	39	0·83 ± 0·03	0·77 ± 0·03	0·09
SDNN				
baseline (ms)	35	24·49 ± 2·11	38·26 ± 7·79	0·08
Stress (ms)	38	26·38 ± 1·94	40·47 ± 7·20	0·14
Recover (ms)	37	27·20 ± 2·28	31·43 ± 4·45	0·70

*Abbreviations: RHR *resting heart rate, *CRT*. chemoradiation therapy, *bpm *beats per minutes, *SBP *systolic blood pressure, *DBP *diastolic blood pressure, *aSKNA *average skin sympathetic nerve activity, *SDNN *standard deviation of normal-to-normal beat intervals, *SE* Standard error

**Fig. 2:**
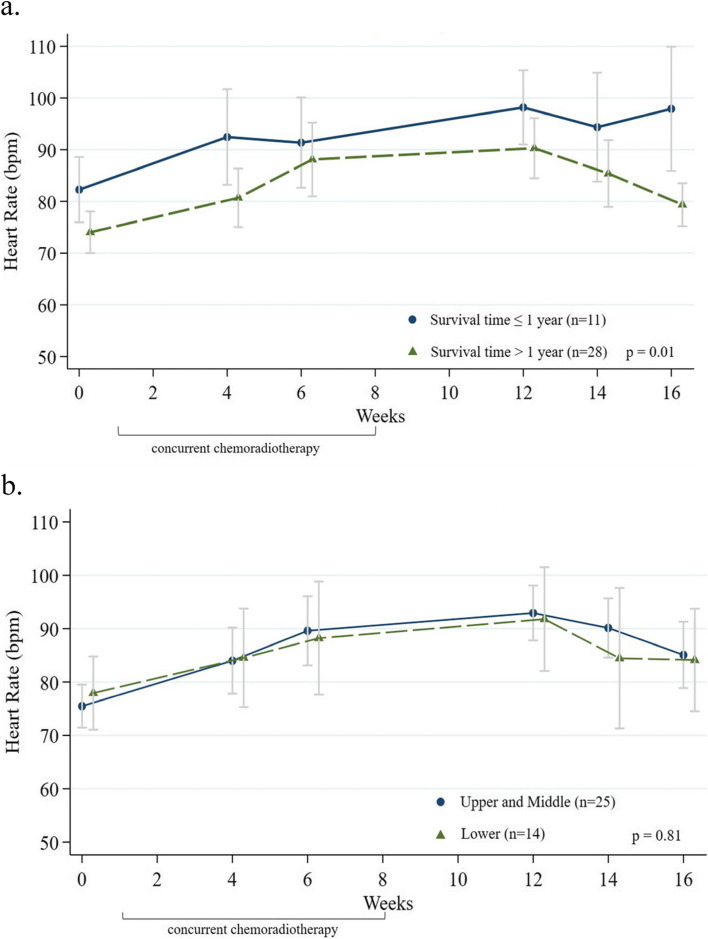
Heart rate change during chemoradiotherapy compared by survival time and primary tumor locations. Abbreviations RHR, Resting heart rate; CRT, chemoradiotherapy **a** RHR changes by time in patients with survival time > 1 year and ≤ 1 year. **b** RHR changes by time in patients with different primary tumor location sites. Week 0 indicated the baseline, before CRT. Patients received CRT from week one to week 8

For the serial RHR change during the 16-week follow-up by GEE model, patients who survived one year or less had significantly elevated RHR after CRT than those surviving more than one year (Fig. 2a, *p* = 0.01); however, there was no significant difference in series RHR change by different tumor site (Fig. 2b) or tumor stage. There was no difference in serial mean arterial pressure (MAP) change between patients from the two survival time periods (Supplementary Fig. 2).

### Predictors for and potentially associated factors with RHR change after CRT

The cutoff point of 18 bpm for difference in serial RHR change from baseline to eight weeks after CRT was obtained by ROC curve. Neither age, gender, pre-treatment NLR, esophagectomy or not, nor the radiation/chemotherapy dose was a significant predictor for RHR change after CRT. The change in RHR was also not significantly related to the change in BMI, albumin, QoL nor the response to CRT (Supplemental Table 1).

### Survival analyses

Eighteen patients died during a median follow up of 11 months (interquartile range 7–14 months). RHR before CRT (crude HR: 1.07, 95% CI: 1.02–1.12, *p* = 0.03) and eight weeks after CRT (crude HR: 1.05, 95% CI: 1.02–1.08, *p* < 0.01), aSKNA before CRT during baseline (crude HR: 9.01, 95% CI: 1.22–66.44, *p* = 0.03) and recovery (crude HR: 19.18, 95% CI: 1.78–206.41, *p* = 0.02) phases were significant risk factors for all-cause mortality in univariate analyses. After adjusting for age, gender, and stage (Table 3), RHR before CRT (adjusted HR: 1.06, 95% CI: 1.01–1.12, *p* = 0.01) and eight weeks after CRT (adjusted HR: 1.05, 95% CI: 1.02–1.08, *p* < 0.01), pre-CRT aSKNA at baseline (adjusted HR: 14.96, 95% CI: 1.19–118.40, *p* = 0.04) and recovery (adjusted HR: 24.89, 95% CI: 1.87–331.11, *p* = 0.02) phases remained significant prognostic factors (Table 3). Higher NLR has been regarded as a predictor of poor prognosis in esophageal cancer [24], although baseline NLR did not have significant correlation with survival in our study. After further adjustment for baseline albumin and NLR, we still found RHR before CRT (adjusted HR: 1.09, 95% CI: 1.02–1.17, *p* = 0.01), RHR eight weeks after CRT (adjusted HR: 1.06, 95% CI: 1.02–1.10, *p* < 0.01), and pre-CRT aSKNA during baseline (adjusted HR: 42.44, 95% CI: 2.41–745.84, *p* = 0.01) and recovery (adjusted HR: 48.77, 95% CI: 2.24–1061.55, *p* = 0.01) phases to be significant predictors for overall survival (Table 3). Response to CRT and the subsequent surgery played an important role in the survival of ESCC patients [25]. After further inclusion of these tow factors, baseline RHR, RHR eight weeks after CRT and pre- CRT aSKNA during baseline and recovery phase remained as significant outcome predictors. The large 95% CI in pre-CRT aSKNA during baseline and recovery phase may be related to our small sample number.

**Table 3:** Association of RHR, aSKNA and SDNN with overall mortality by Cox regression analysis

	Hazard ratio (95%CI)	*p* value	Adjusted hazard ratio^a^ (95%CI)	*p* value	Adjusted hazard ratio^b^ (95%CI)	*p* value	Adjusted hazard ratio^c^ (95%CI)	*p* value
RHR before CRT	1·07(1·02–1·12)	0·03	1·06(1·01–1·12)	0·01	1·09 (1·02–1·17)	0·01	1.12 (1.03–1.21)	0.01
RHR 4 weeks after CRT	1·02(0·99–1·05)	0·16	1·04(1·00–1·07)	0·04	1·04 (1·00–1·08)	0·04	1.05 (1.01–1.09)	0.02
RHR 8 weeks after CRT	1·05(1·02–1·08)	< 0·01	1·05(1·02–1·08)	< 0·01	1·06 (1·02–1·10)	< 0·01	1.05 (1.01–1.09)	0.01
aSKNA before CRT								
baseline	9·01(1·22–66·44)	0·03	14·96(1·19–118·40)	0·04	42·44 (2·41–745·84)	0·01	406.14 (15.40–10,707.44)	< 0.01
stress	1·92(0·91–4·05)	0·09	1·91(0·83–4·41)	0·13	2·03 (0·83–4·97)	0·12	34.06 (5.64–205.59)	< 0.01
recover	19·18 (1·78–206·41)	0·02	24·89(1·87–331·11)	0·02	48·77 (2·24–1061·55)	0·01	4267.85(57.9–314,212.4)	< 0.01
SDNN before CRT								
baseline	0·99(0·95–1·03)	0·55	1·00(0·95–1·05)	0·87	0·98 (0·93–1·04)	0·51	0.96 (0.90–1.02)	0.18
stress	0·98(0·94–1·02)	0·32	0·99(0·95–1·03)	0·64	0·98 (0·94–1·02)	0·35	0.97 (0.92–1.01)	0.17
recovery	0·97(0·93–1·01)	0·15	0·97(0·93–1·01)	0·12	0·95 (0·91–0·99)	0·05	0.94 (0.89–0.99)	0.03
aSKNA after CRT								
baseline	3·02(0·28–32·30)	0·36	6·23(0·67–58·38)	0·11	9·32 (0·90–96·38)	0·06	16.89 (1.23–232.22)	0.04
stress	1·34(0·54–3·30)	0·52	1·67(0·45–6·27)	0·45	1·23 (0·32–4·75)	0·77	13.83 (1.48–129.57)	0.02
recover	1·31(0·10–17·89)	0·84	3·16(0·20–50·41)	0·42	2·98 (0·14–62·57)	0·48	83.63 (2.01–3475.36)	0.02
SDNN after CRT			0.06(0.00–52.48)					
baseline	1·00(0·99–1·02)	0·72	1·01(0·99–1·02)	0·35	1·01 (0·99–1·02)	0·45	1.01 (0.99–1.02)	0.42
stress	1·00(0·99–1·00)	0·95	1.00(0·99–1·01)	0·76	1·00 (0·99–1·01)	0·56	1.02 (1.00–1.04)	0.06
recovery	0·98(0·96–1·01)	0·18	0·98(0·95–1·01)	0·16	0·95 (0·91–0·99)	0·03	0.95 (0.91–0.99)	0.02

*Abbreviations: RHR *resting heart rate, *aSKNA *average skin sympathetic nerve activity, *SDNN *standard deviation of normal-to-normal beat intervals, *CRT* chemoradiotherapy, *CI* Confidence interval

^a^adjusted for age, gender, and stage

^b^adjusted for age, gender, stage, baseline albumin level and neutrophil-to-lymphocyte ratio (NLR)

^c^adjusted for age, gender, stage, baseline albumin level, neutrophil-to-lymphocyte ratio (NLR) and CRT response (partial response vs. stable disease + progressive disease), operation or not

**Fig. 3:**
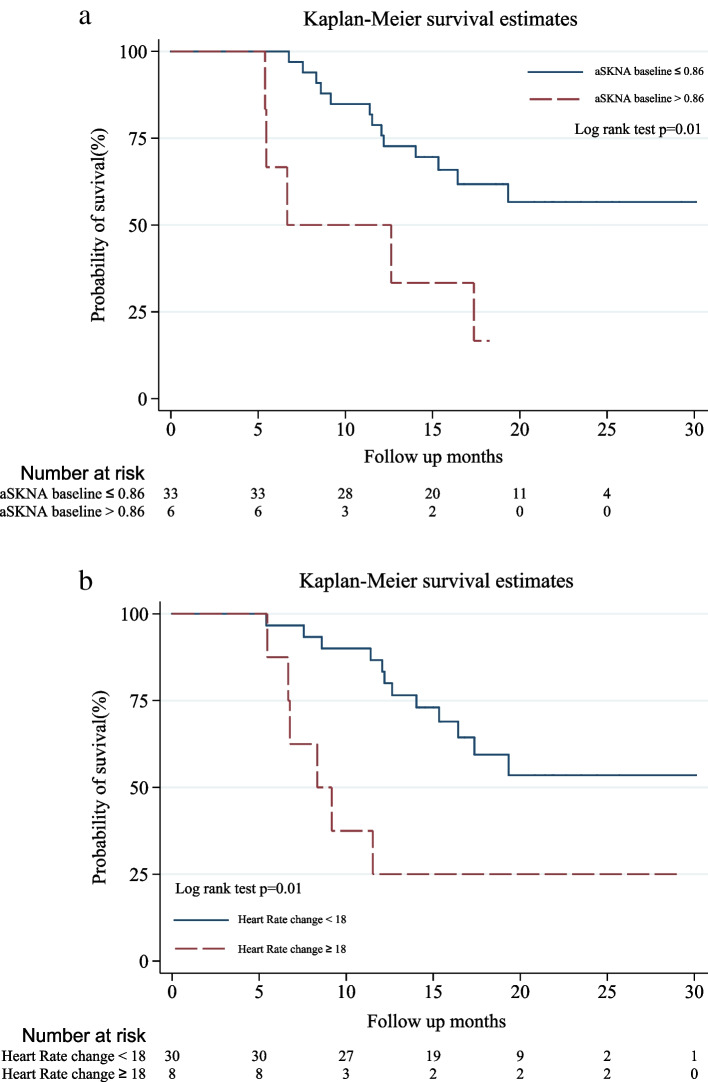
Elevated resting heart rate after chemoradiotherapy and higher baseline sympathetic tone were related to worse overall survival Abbreviation aSKNA: average skin sympathetic nerve activity, RHR: resting heart rate. a. Overall survival rates by changes in RHR (< 18 or ≥ 18 bpm; *p* = 0.01) b. Overall survival rates by aSKNA (≤ 0.86 or > 0.86; *p* = 0.01). The cutoff point of RHR and aSKNA were obtained by ROC curve

Kaplan–Meier survival curves showed patients with elevated RHR above the cutoff level (18 bpm) after CRT (Fig. 3a) had worse overall survival. Meanwhile, those with higher baseline sympathetic tone (aSKNA cutoff level by ROC curve ≥ 0.86 μV) were also at higher risk of death (Fig. 3b). As for the parasympathetic tone measured by SDNN, we used the cut-off values 10 ms [26] and 20 ms [27] which have been reported to predict the prognosis in cancer patients. However, we did not find significant association between baseline SDNN and the outcome of our patients.

## Discussion

In this carefully designed cohort study using the novel neoECG to harvest patients’ autonomic activity change, we confirmed elevation of RHR is a common phenomenon in ESCC patients undergoing CRT despite best supportive care to avoid dehydration and to control pain. Those with persistently elevated RHR at the eighth week after CRT had higher mortality rate. One mechanism behind the phenotype RHR could be increased sympathetic tone by the baseline and recovery phase of pre-treatment SKNA. Moreover, we found elevated RHR and higher pre-treatment SKNA were significant predictors of overall survival in ESCC patients undergoing CRT.

Previous studies have reported increased RHR and decreased blood pressure after CRT in esophageal cancer patients [28–31]. Mohammad et al. concluded these phenomena were simply related to dehydration [30]. However, Hatakenaka et al. considered that instead of dehydration, elevated RHR compensated the reduced left ventricular stroke volume index related to CRT [28]. Zhang et al. proposed radiotherapy-induced conduction system abnormality may be one etiology [31], as radiation could damage the heart and cause autonomic dysfunction, leading to elevated RHR and abnormal heart rate recovery [11]. Moreover, stimulation to left stellate ganglion plays an important role in cardiac arrhythmogenesis [32]. However, we did not find a significantly higher RHR after CRT in upper- and mid-third ESCC patients in whom the radiation field covered the neck, including the stellate ganglion. The exact mechanisms behind elevated RHR after CRT including autonomic dysfunction and cardiotoxicity warrant further investigation.

Elevated RHR has been linked to all-cause mortality, cardiovascular mortality, and cancer mortality [1–5]. In our study, the RHR before and after CRT was also associated with ESCC mortality. Mohammad et al. showed that the RHR at the end of CRT was significantly higher than that before CRT [30]. Gradually increased RHR measures from baseline to end of CRT or before [29], during and after CRT have been reported [28], but only demonstrating short-term change (till 0–12 days after completion of CRT) and the finding was not translated into outcome significance. Our study presented a closer and longer monitoring period showing a gradually increased mean RHR, peaking in the fourth weeks after CRT and then gradually decreasing but remaining higher than baseline. Those without recovery of elevated RHR had worse outcome. Abnormal heart rate recovery after exercise is an established predictor of autonomic dysfunction [11], and since exercise and CRT both affect autonomic nerve system, we suggest persistently elevated RHR eight weeks after completion of CRT might indicate prolonged autonomic dysfunction after treatment, hence worse outcome.

We found baseline sympathetic nerve activity as indexed by aSKNA but not HRV was related to ESCC survival. To the best of our knowledge, this is the first study to demonstrate higher baseline aSKNA as a predictor of ESCC mortality. Along with our study, previous studies have shown that higher sympathetic nerve activity could promote cancer initiation, metastasis, and hence mortality in cancer patients [27]. On the other hand, cancers might promote their growth and survival by reactivating nerve-dependent developmental and regenerative processes [33]. The nerve-cancer cross-talk is also important for tumor progression and provides the basis for effective targets for the inhibition of tumor-induced neurogenesis and tumor progression [34]. Thus, estimating the sympathetic nerve status is very important for cancer survey. neuECG is a novel and promising tool to noninvasively measure sympathetic nerve activity as it can estimate stellate ganglion activity, which represents the cardiac sympathetic tone [35]. Increased SKNA has been shown to proceed the onset and termination of atrial tachycardia [36]. Huang et al. showed the resting SKNA could predict syncope episode [35]. Moreover, a good correlation between SKNA and heart rate has been reported in patients of head tilt-up positive patients and paroxysmal atrial arrhythmia patients [35, 36], although we did not find such correlation in ESCC patients. It is possible that other factors such as cardiovascular function or tumor burden also play a role on RHR.

HRV measures RR interval and represent a complex interaction between sympathetic and parasympathetic influence on the sinoatrial node [9]. Several factors may influence HRV, such as sinoatrial node function, respiratory sinus arrhythmia, endocrine system, immune system, metabolic system, psychological function, etc. [37]. Therefore, there is an interrelationship between HRV and autonomic nerve systems. In contrast to our observation, higher vagal nerve activity, indexed by HRV, has been linked to better outcome in patients with breast cancer, pancreatic cancer, and non-small-cell lung cancer, etc. [27, 38–40]. The discrepancy may come from sex ratio, different methods and measurement duration of acquiring HRV, cancer types, chemotherapy regimens, radiation exposure area, etc. [39]. Although some researchers regard 24-h SDNN as the “gold standard”, it is expensive, complex, time-consuming, and inconvenient for patients [12, 41]. The method we utilized, 5-min SDNN, was therefore widely adopted in clinical studies [39]. Compared to previous studies, our patients were male-dominant (92.31%). In a Korean study, men had significantly lower SDNN than women [42]. Another meta-analysis focusing on sex difference in HRV indicated that men had significantly higher SDNN values [43]. Despite the inconsistency, these studies suggest that gender can affect SDNN values. Hence, previous SDNN cut-off values may not be applicable to our male-dominant population.

Stellate ganglion is a sympathetic ganglion and is an importance source of cardiac sympathetic innervation [32]. Direct recording of stellate ganglion nerve activity (SGNA) was thought to be as a “gold standard” to represent the cardiac sympathetic outflow [19]. In addition, stellate ganglion also innervate the skin of upper thorax and extremities, thus using SKNA measurement from the skin of upper chest wall to estimate SGNA is reasonable [32, 36]. According to Chan et al., subcutaneous nerve activity (SCNA) is more accurate than HRV in estimating cardiac sympathetic tone [19]. Furthermore, SKNA has been shown to correlate strongly with SCNA and SGNA as well [32]. Since there is no standard methods to analyze HRV and multiple confounders should be considered in interpreting HRV data [37, 39], SKNA might be a better method to evaluate autonomic system function especially the sympathetic part. On the other hand, the radiation therapy of ESCC patients might affect the sinoatrial node function [44], thus using HRV, a sinoatrial function dependent examination, to estimate the autonomic function in ESCC patients might be inaccurate. Therefore, other than using HRV, using SKNA to estimate autonomic function in ESCC patients should be a better alternative.

There are several limitations in this study. Firstly, there were only 39 patients and we recorded RHR and blood pressure till 8 weeks after completion of CRT. However, this is a novel and careful study of highly selected participants to minimize the effects of patient comorbidity, performance, and treatment side effects on serial heart rate change. Secondly, neuECG uses traditional ECG electrodes to harvest electrical signals from the skin, and in contact between skin and electrodes, motion artifact and room temperature could affect the results of our measurement, but to avoid such interference, the dirt, hair, oils, and desquamations of skin were cleaned before recording; subjects were asked to be still during the recording; and the room temperature and moisture were fixed for each subject.

In conclusion, our findings suggest elevated RHR after CRT is an alarm sign of poor ESCC outcome. Increased pre-treatment SKNA may also predict worse survival. Further study is necessary to investigate the detailed mechanism behind this phenomenon and to find potential interventions able to avoid this. Clinical trials on the reversal effect using autonomic modulation drugs that significantly reduce heart rate, such as beta-blocker and I_f_-inhibitor (ivabradine), may be conducted in ESCC patients undergoing CCRT especially those with a higher baseline SKNA.

## Supplementary Information

**Additional file 1: Supplementary Figure 1.** treatment protocol and the timing of resting heart rate, blood pressure and neuECG.

**Additional file 2: Supplementary Figure 2.** Mean arterial pressure (MAP) change by time in 2 groups based on survival time >1 year and ≤ 1 year.

**Additional file 3: Supplementary Table 1.** Factors potentially associated with heart rate change1 after CRT.

## Data Availability

The datasets used and analyzed during the current study are available from the corresponding author on reasonable request.

## Abbreviations

RHR: Resting heart rate
SKNA: Skin sympathetic nerve activity
CRT: Chemoradiotherapy
ESCC: Esophageal squamous cell carcinoma
ECOG: Eastern Cooperative Oncology Group
IRB: Institutional Review Board
EGD: Esophagogastroduodenoscopy
CT: Computer tomography
HRV: Heart rate variability
aSKNA: Average skin sympathetic nerve activity
SDNN: Standard deviation of normal-to-normal beat interval
EORTC: European Organization for Research and Treatment for Cancer
SD: Standard deviation
QoL: Quality of life
CI: Confidence interval
GEE: Generalized estimating equation
BMI: Body mass index
NLR: Neutrophil-to-lymphocyte ration
MAP: Mean arterial pressure
SGNA: Stellate ganglion nerve activity
SCNA: Subcutaneous nerve activity
