# Supplementary material for: Higher pre-treatment skin sympathetic nerve activity and elevated resting heart rate after chemoradiotherapy predict worse esophageal cancer outcomes
Source: BMC Cancer. 2022 Oct 22;22:1086. doi: 10.1186/s12885-022-10180-8 (PMC9587625; doi:10.1186/s12885-022-10180-8)
Supplement: Supplementary file 1 — Additional file 1: Supplementary Figure 1. treatment protocol and the timing of resting heart rate, blood pressure and neuECG. [file 12885_2022_10180_MOESM1_ESM.pdf]

Supplementary Figure 1. treatment protocol and the timing of resting heart rate, blood pressure and neuECG.

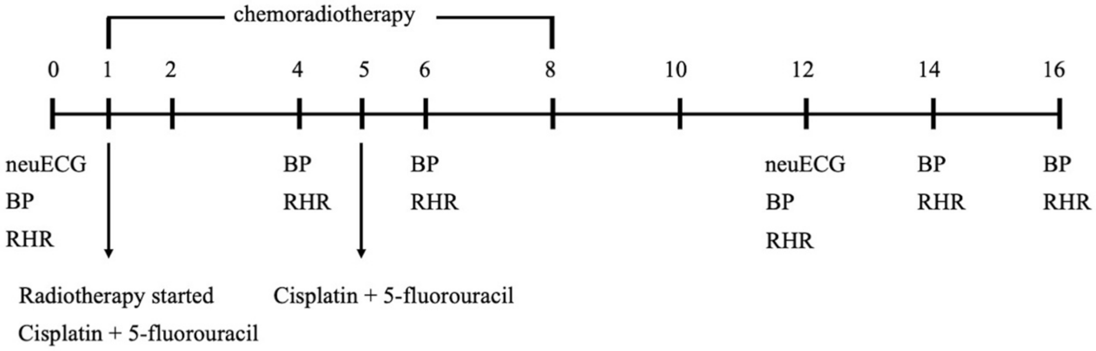

Abbreviation: BP: blood pressure, RHR: resting heart rate, CRT: chemoradiotherapy
