# Supplementary material for: Higher pre-treatment skin sympathetic nerve activity and elevated resting heart rate after chemoradiotherapy predict worse esophageal cancer outcomes
Source: BMC Cancer. 2022 Oct 22;22:1086. doi: 10.1186/s12885-022-10180-8 (PMC9587625; doi:10.1186/s12885-022-10180-8)
Supplement: Supplementary file 2 — Additional file 2: Supplementary Figure 2. Mean arterial pressure (MAP) change by time in 2 groups based on survival time >1 year and ≤ 1 year. [file 12885_2022_10180_MOESM2_ESM.pdf]

Supplementary Figure 2. Mean arterial pressure (MAP) change by time in 2 groups based on survival time >1 year and ≤ 1 year.

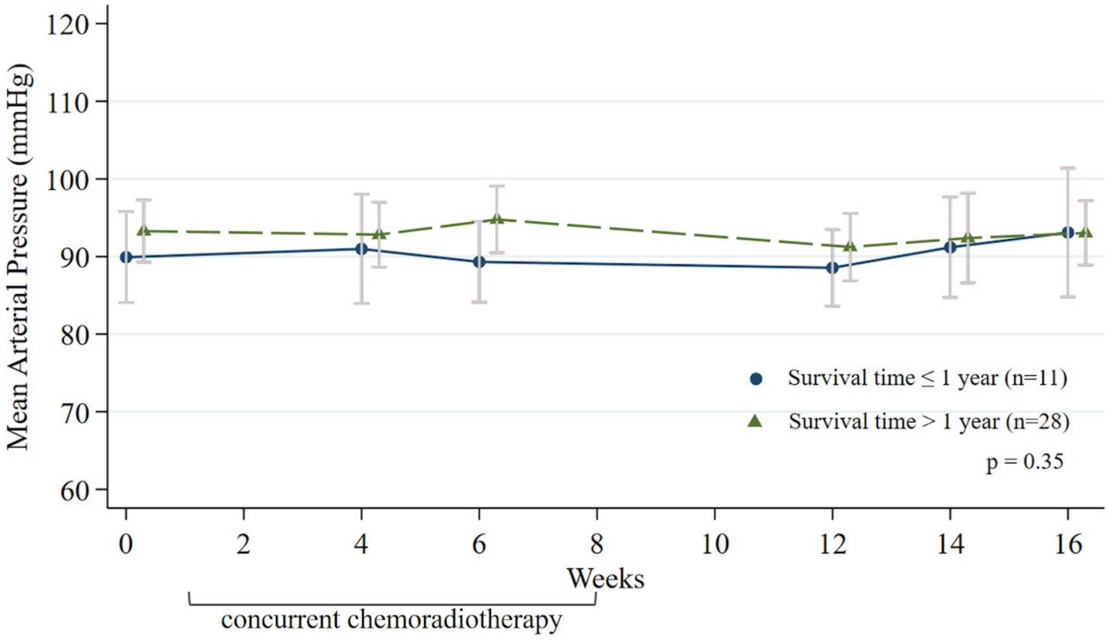

Abbreviation: aSKNA: average skin sympathetic nerve activity, RHR: resting heart rate, CRT: chemoradiotherapy

Patient started CRT at week 1 and end in week 8. Patient received post CRT autonomic evaluation on week 12 (4 week after CRT).
