# Supplementary material for: Higher pre-treatment skin sympathetic nerve activity and elevated resting heart rate after chemoradiotherapy predict worse esophageal cancer outcomes
Source: BMC Cancer. 2022 Oct 22;22:1086. doi: 10.1186/s12885-022-10180-8 (PMC9587625; doi:10.1186/s12885-022-10180-8)
Supplement: Supplementary file 3 — Additional file 3: Supplementary Table 1. Factors potentially associated with heart rate change1 after CRT. [file 12885_2022_10180_MOESM3_ESM.pdf]

Supplementary Table 1. Factors potentially associated with heart rate change[1] after CRT

	Heart rate change[1] < 18bpm (n=30)	Heart rate change[1] ≥ 18 bpm (n=8)	
Variable	N (%)	N (%)	p value
Age, years (mean ± SD)	59·33 ± 6·50	58·63 ± 7·61	0·79
< 59	14 (46·67%)	3 (37·50%)	
≥ 59	16 (53·33%)	5 (62·50%)	
Sex			0·52
Female	2 (6·76%)	1 (13·50%)	
Male	28 (93·33%)	7 (87·50%)	
Operation			0·11
Yes	15 (50·00%)	1 (12·50%)	
No	15 (50·00%)	7 (87·50%)	
BMI change			0·68
< -0·64	14 (46·67%)	4 (50·00%)	
≥ -0·64	16 (53·33%)	3 (37·50%)	
missing	0 (0·00%)	1 (12.50%)	
Albumin change (g/dL)			0·66
< -0·09	14 (46·67%)	4 (50·00%)	
≥ -0·09	16 (53·33%)	2 (25·00%)	
missing	0 (0·00%)	2 (25·00%)	
EORTC QLQ-C30 score change[2]			0·73
< 2·38	15 (50·00%)	3 (37·50%)	
≥ 2·38	15 (50·00%)	4 (50·00%)	
missing	0 (0·00%)	1 (12·50%)	
EORTC QLQ-OES18 score change[2]			0·41
< 1·67	14 (46·67%)	5 (62·50%)	
≥ 1·67	16 (53·33%)	2 (25·00%)	
missing	0 (0·00%)	1 (12·50%)	
CRT response			0·33
Partial response	25 (83·33%)	5 (62·50%)	
Stable and progressive disease	5 (16·67%)	3 (37·50%)	

Abbreviations: CRT, chemoradiotherapy; BMI, body mass index; EORTC QLQ C-30, European Organization for Research and Treatment of Cancer quality of life questionnaire; EORTC QLQ-OES18, European Organization for Research and Treatment of Cancer quality of life questionnaire for oesophageal cancer; SD, standard deviation

1. ROC curve cut point of difference between the heart rate before CRT and at 8 weeks after CRT (16th week). Heart rate change, Mean ± SD: 8·05 ± 15·38

2. The cut-off value of EORTC QLQ-C30 and QLQ-OES18 was obtained from the maximum Youden's index of their ROC curve.
